# Structural snapshot of a glycoside hydrolase family 8 endo-β-1,4-glucanase capturing the state after cleavage of the scissile bond

**DOI:** 10.1107/S2059798321012882

**Published:** 2022-01-24

**Authors:** Takaaki Fujiwara, Ayumi Fujishima, Yui Nakamura, Kenji Tajima, Min Yao

**Affiliations:** a Faculty of Advanced Life Science, Hokkaido University, Kita-10, Nishi-8, Kita-ku, Sapporo 060-0180, Japan; b Graduate School of Life Science, Hokkaido University, Kita-10, Nishi-8, Kita-ku, Sapporo 060-0180, Japan; c Faculty of Engineering, Hokkaido University, Kita-13, Nishi-8, Kita-ku, Sapporo 060-8628, Japan

**Keywords:** bacterial cellulose, crystal structure, intermediates, hydrolysis, BcsZ, endo-β-1,4-glucanase

## Abstract

Bacterial cellulose (BC), which is produced by bacteria, is a biodegradable and biocompatible natural resource. Because of its remarkable physicochemical properties, BC has attracted attention for the development and manufacture of biomedical and industrial materials. In the BC production system, the enzyme endo-β-1,4-glucanase, which belongs to glycoside hydrolase family 8 (GH8), acts as a cleaner by trimming disordered cellulose fibers to produce high-quality BC. Understanding the molecular mechanism of the endo-β-1,4-glucanase would help in developing a reasonable biosynthesis of BC. Nevertheless, all of the steps in the reaction of this endo-β-1,4-glucanase are not clear. This study confirms the BC hydrolytic activity of the endo-β-1,4-glucanase from the BC-producing bacterium *Enterobacter* sp. CJF-002 (*Eb*BcsZ) and reports crystal structures of *Eb*BcsZ. Unlike in previously reported GH8 endo-β-1,4-glucanase structures, here the base catalyst was mutated (D242A) and the structure of this mutant bound to cellooligosaccharide [*Eb*BcsZ(D242A)_CPT_] was analyzed. The *Eb*BcsZ(D242A)_CPT_ structure showed two cellooligosaccharides individually bound to the plus and minus subsites of *Eb*BcsZ. The glucosyl unit in subsite −1 presented a distorted ^5^ *S*_1_ conformation, a novel snapshot of a state immediately after scissile-bond cleavage. In combination with previous studies, the reaction process of endo-β-1,4-glucanase is described and the β-1,4-glucan-trimming mechanism of *Eb*BcsZ is proposed. The *Eb*BcsZ(D242A)_CPT_ structure also showed an additional β-1,4-glucan binding site on the *Eb*BcsZ surface, which may help to accept the substrate.

## Abstract 2

The structure of an endo-β-1,4-glucanase from the cellulose-producing bacterium *Enterobacter* sp. CJF-002 bound to cellooligosaccharide was determined.

## 1. Introduction

Worldwide, cellulose (β-1,4-glucan) production mainly relies on plants, but some bacteria can also produce a type of cellulose named bacterial cellulose (BC; Somerville, 2006 ▸; Ross *et al.*, 1991 ▸). Cellulose-producing bacteria secrete BC as an exopolysaccharide, forming a hierarchical assembly outside the cell. Unlike the lignocellulosic matrix derived from plant cell walls, BC displays a naturally high purity and high crystallinity (Chundawat *et al.*, 2011 ▸; Czaja *et al.*, 2004 ▸). BC also has high mechanical strength (Yamanaka *et al.*, 1989 ▸), bio­degradability (Torgbo & Sukyai, 2020 ▸) and biocompatibility (Helenius *et al.*, 2006 ▸). These unique characteristics make BC an attractive environmentally friendly resource for the development and manufacture of biomedical or industrial materials such as wound dressings (Czaja *et al.*, 2006 ▸), artificial blood vessels (Lee & Park, 2017 ▸), electronic paper displays (Nogi & Yano, 2008 ▸; Shah & Brown, 2005 ▸) and speaker diaphragms (Nishi *et al.*, 1990 ▸). Despite numerous efforts to improve BC production (Lee *et al.*, 2014 ▸), such as optimization of the composition of culture media, bioreactors and bacterial strains, bacteria do not satisfy the demand for BC. Understanding the mechanism of BC biosynthesis will therefore help to produce BC more efficiently, and has become a hot topic in BC research.

Cellulose-producing bacteria biosynthesize BC using a multi-protein complex (terminal complex; TC) localized on the outer and inner membranes. *Komagataeibacter xylinus* (formerly called *Acetobacter xylinum*) serves as a model bacterium for BC biosynthesis. In *K. xylinus*, the TC is composed of BC synthase subunits A, B, C and D (BcsA, BcsB, BcsC and BcsD, or alternatively BcsAB, BcsC and BcsD; Kawano *et al.*, 2002 ▸; Wong *et al.*, 1990 ▸). Analyzing the individual structures of the subunits clarified their roles in BC biosynthesis (Acheson *et al.*, 2019 ▸; Nojima *et al.*, 2017 ▸; Fujiwara *et al.*, 2013 ▸; Morgan *et al.*, 2013 ▸, 2016 ▸; Hu *et al.*, 2010 ▸). The production of high-quality BC depends on both the TC and some accessory factors such as cleaner proteins. The *cmcax* gene, which is located upstream of the BC synthase operon in *K. xylinus*, encodes CMCax, an endo-β-1,4-glucanase (EC 3.2.1.4) that hydrolyzes carboxymethyl cellulose (CMC) and cellooligosaccharides (Yasutake *et al.*, 2006 ▸; Standal *et al.*, 1994 ▸). Disruption of the *cmcax* gene reduced the amount of BC produced (Nakai *et al.*, 2013 ▸), while overexpression of CMCax increased it (Kawano *et al.*, 2008 ▸). Furthermore, adding a CMCax antibody to the culture medium inhibited the formation of BC fibers (Koo *et al.*, 1998 ▸). Considering the hydrolytic activity of CMCax, endo-β-1,4-glucanase could act as a cleaner protein to produce high-quality BC by trimming disordered cellulose. Interestingly, BcsZ, an endo-β-1,4-glucanase encoded in the BC synthase operon, also exists in most bacterial lineages (Römling & Galperin, 2015 ▸). Recent studies showed that *Enterobacter* sp. CJF-002, isolated from rocks in oil reservoirs, efficiently produces BC and is a promising bacterium for producing large amounts of BC (Sunagawa *et al.*, 2012 ▸). In *Enterobacter* sp. CJF-002, a single operon encodes BcsA, BcsB, BcsZ and BcsC. Although CMCax and BcsZ have low sequence similarity (19% identity), the residues located in the catalytic site are highly conserved (Supplementary Fig. S1). BcsZ from *Enterobacter* sp. CJF-002 (*Eb*BcsZ; GenBank accession No. BAM44856) and cellulolytic *Escherichia coli* (*Ec*BcsZ) also hydrolyze cellooligosaccharides and CMC (Pang *et al.*, 2019 ▸; Sunagawa *et al.*, 2012 ▸; Mazur & Zimmer, 2011 ▸), indicating that BcsZ and CMCax are functionally homologous. Therefore, a biological function of *Ec*BcsZ could be to promote the formation of cellulose fibers by degrading β-1,4-glucan chains (Mazur & Zimmer, 2011 ▸).

**Figure 1:**
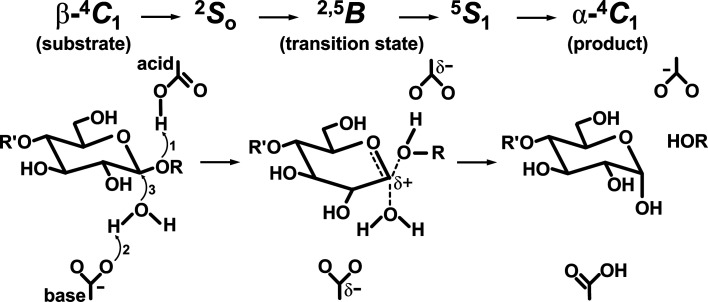
General reaction mechanism of inverting glycoside hydrolases. The upper part shows the proposed conformational changes of the sugar in subsite −1 (G_−1_). β-^4^ *C*_1_, ^2,5^ *B* and α-^4^ *C*_1_ are the conformations of G_−1_ in the substrate, oxocarbenium ion intermediate (transition state) and product, respectively. ^2^ *S*_O_ and ^5^ *S*_1_ are the conformations of the other intermediates. The lower part shows the proposed catalytic process of GH8 enzymes. The reaction follows the single-displacement mechanism through the oxocarbenium ion intermediate. The numbers in the left part represent the order of the displacement reaction, *i.e.* protonation of the glycosidic bond occurs followed by nucleophilic attack of an activated water molecule. G_−1_ is shown as a six-membered sugar. The substrate, transition intermediate and product are shown in the left, middle and right panels, respectively. Before and after forming the oxocarbenium intermediate, G_−1_ forms ^2^ *S*_O_ and ^5^ *S*_1_ conformations, respectively.

Similar to endo-β-1,4-xylanases (EC 3.2.1.8), licheninases (EC 3.2.1.73), chitosanases (EC 3.2.1.132) and reducing-end xylose-releasing exo-oligoxylanases (EC 3.2.1.156), CMCax and BcsZ belong to glycoside hydrolase family 8 (GH8; CAZy; http://www.cazy.org). The catalytic reaction of GH8 enzymes is a single-displacement mechanism accompanied by an inversion of the anomeric configuration of the sugar unit in subsite −1, which changes the conformation of pyranose from β-^4^ *C*_1_ to α-^4^ *C*_1_ (Koshland, 1953 ▸; Cremer & Pople, 1975 ▸; Fig. 1 ▸, Supplementary Fig. S1). The general acid catalyst donates a proton to the C1 atom to release the hemiacetal sugar. The general base catalyst activates a water molecule for a nucleophilic attack on the anomeric center (C1) of the substrate. Displacement of the activated water molecule and liberation of the leaving group of the product occur on the C1 atom of a distorted oxocarbenium ion intermediate, forming an α-configuration at the C1 atom. To understand the reaction mechanism of GH8 enzymes, structures of GH8 enzymes and their acid catalyst mutants bound to oligosaccharides have been analyzed to date. In the structure of the E55Q mutant of *Ec*BcsZ bound to cellopentaose (CPT) [*Ec*BcsZ(E55Q)_CPT_; PDB entry 3qxq], the glucosyl unit in subsite −1 (G_−1_) presented an α-^4^ *C*_1_ conformation, showing that *Ec*BcsZ(E55Q)_CPT_ is the product-binding form (Mazur & Zimmer, 2011 ▸). In contrast, the structure of the E95Q mutant of CelA from *Clostridium thermocellum* bound to CPT [*Ct*CelA(E95Q)_CPT_; PDB entry 1kwf] showed that G_−1_ presented a ^2,5^ *B* conformation and thus *Ct*CelA(E95Q)_CPT_ was an intermediate in the transition state (Guérin *et al.*, 2002 ▸). Interestingly, the structure of wild-type endo-β-1,4-xylanase from *Teredinibacter turnerae* bound to xylotriose (XTO) (*Tt*GH8_XTO_; PDB entry 6g0b) confirmed the existence of a ^2,5^ *B* conformation of the xylosyl unit in subsite −1 (X_−1_; Fowler *et al.*, 2018 ▸). Based on these structures, a computational simulation predicted that in the reaction process of GH8 enzymes the G_−1_ conformational change sequence was β-^4^ *C*_1_→^2^ *S*_O_→^2,5^ *B*→^5^ *S*_1_→α-^4^ *C*_1_ (Ardèvol & Rovira, 2015 ▸; Petersen *et al.*, 2009 ▸; Fig. 1 ▸). However, the coordinates of the ^2^ *S*_O_ and ^5^ *S*_1_ intermediates remain unclear since no structural data are available to prove the existence of such intermediates of GH8 enzymes.

In this study, we assessed the hydrolytic activity of *Eb*BcsZ on BC produced by *K. xylinus*. Based on the idea that BcsZ hydrolyzes cellulose through an inverting mechanism, we mutated the base catalyst Asp242 to alanine (D242A) and determined the structure of this mutant in complex with a cellooligosaccharide [*Eb*BcsZ(D242A)_CPT_]. The *Eb*BcsZ(D242A)_CPT_ structure showed a novel snapshot of a reaction stage immediately after scissile-bond cleavage. It displayed a distorted ^5^ *S*_1_ conformation of G_−1_. Moreover, the structure of wild-type *Eb*BcsZ in complex with glycerol (*Eb*BcsZ_GOL_) supported the existence of the ^5^ *S*_1_ conformation in the reaction process of BcsZ. We finally compared our structures with those of other GH8 endo-β-1,4-glucanases, extended the previously described reaction mechanism of GH8 enzymes and proposed a β-1,4-glucan-trimming mechanism for *Eb*BcsZ.

## 2. Materials and methods

### 2.1. Cloning, expression and purification

Except for the N-terminal signal peptide (Met1–Ala20), we amplified the gene fragment encoding *Eb*BcsZ (amino acids Ala21–Gln367) by PCR using the genome of *Enterobacter* sp. CJF-002 as a template using the primers 5′-GGAATTCCATATGGCCTGCACATGGCCTGC-3′ and 5′-CCGCTCGAGTTACTGTGAACTTGCGCATGCCTG-3′. We used NdeI and XhoI to digest the amplified gene fragments and the protein expression vector pET-28b (Novagen) with a hexahistidine tag and thrombin cleavage sequence at the N-terminus, and then ligated them. We transformed the expression plasmids into *E. coli* strain BL21(DE3) by electroporation. We grew the cells in LB medium supplemented with 25 µg ml^−1^ kanamycin at 37°C until the OD_600_ reached 0.5. We then induced overexpression of recombinant *Eb*BcsZ by adding 100 µ*M* isopropyl β-d-1-thiogalactopyranoside to the culture medium and cultured the cells overnight at 25°C. After resuspending the harvested cells in buffer *A* [50 m*M* HEPES–KOH buffer pH 8.0 containing 500 m*M* potassium chloride and 10%(*v*/*v*) glycerol], we lyzed the cells by sonication. We centrifuged the cell lysate at 40 000*g* for 15 min at 10°C and then loaded the supernatant onto a nickel-affinity chromatography column (5 ml HisTrap HP; Cytiva) equilibrated with buffer *A*. We then eluted the bound *Eb*BcsZ with buffer *B* [50 m*M* HEPES–KOH buffer pH 8.0 containing 500 m*M* potassium chloride, 10%(*v*/*v*) glycerol and 500 m*M* imidazole] in a linear gradient of imidazole from 50 to 500 m*M*. We collected the eluate containing *Eb*BcsZ and then removed the N-terminal hexahistidine tag using thrombin (Fujifilm Wako) overnight at 4°C. We then again loaded *Eb*BcsZ onto a nickel-affinity chromatography column and collected the eluate containing the protein, which had lost its affinity for the column. Finally, we purified *Eb*BcsZ on a Superdex 200 16/600 column (Cytiva) equilibrated with buffer *C* (50 m*M* HEPES–KOH buffer pH 8.0 containing 150 m*M* potassium chloride). We introduced the D242A mutation using the designed primers by the QuikChange technique and confirmed it by sequencing. We prepared the *Eb*BcsZ(D242A) mutant as described above for wild-type *Eb*BcsZ. We concentrated the purified *Eb*BcsZ and *Eb*BcsZ(D242A) to a final concentration of 9 mg ml^−1^ by ultrafiltration using Vivaspin 6 concentators with a 10 kDa cutoff membrane (Cytiva).

### 2.2. Crystallization and X-ray diffraction data collection

We performed initial crystallization screening for both proteins using sparse-matrix crystallization kits from Qiagen, Hilden, Germany. We mixed 0.5 µl protein solution with an equal volume of reservoir solution and used the sitting-drop vapor-diffusion method. We obtained *Eb*BcsZ and *Eb*BcsZ(D242A) crystals using the same reservoir solution consisting of 0.2 *M* disodium tartrate, 20%(*w*/*v*) PEG 3350. Crystals grew to dimensions of 0.3 × 0.2 × 0.2 mm within three days at 20°C. We transferred the *Eb*BcsZ(D242A) crystals into soaking solution [0.2 *M* disodium tartrate, 20%(*v*/*v*) PEG 3350, 20%(*v*/*v*) glycerol, 2 m*M* CPT] and incubated them for 20 h at 20°C. We soaked the *Eb*BcsZ crystals in reservoir solution supplemented with 20%(*v*/*v*) glycerol and then flash-cooled both crystals under a stream of liquid nitrogen. We collected diffraction data on beamlines NW-12A at Photon Factory, Tsukuba, Japan and BL41XU at SPring-8, Hyogo, Japan and processed the data using *XDS* (Kabsch, 2010 ▸). Table 1 ▸ summarizes the data statistics.

### 2.3. Structure determination and refinement

To determine the structure of *Eb*BcsZ in complex with glycerol (*Eb*BcsZ_GOL_), we used the molecular-replacement (MR) method with *AutoMR* from the *Phenix* package (Liebschner *et al.*, 2019 ▸) using the *Ec*BcsZ_apo_ structure (PDB entry 3qxf; Mazur & Zimmer, 2011 ▸) as the search model. We calculated the rotation and translation functions using data in the resolution range 45.0–3.0 Å. Similarly, we determined the *Eb*BcsZ(D242A)_CPT_ structure by the MR method with *AutoMR* using the *Eb*BcsZ_GOL_ structure as the search model. We calculated rotation and translation functions using data in the resolution range 45.0–2.5 Å. We performed several rounds of refinement using *phenix.refine* from the *Phenix* suite, alternating with manual fitting and rebuilding based on 2*F*_o_ − *F*_c_ and *F*_o_ − *F*_c_ electron-density maps using *Coot* (Emsley *et al.*, 2010 ▸). We confirmed the conformation of the β-1,4-glucan chains based on the omit map. Table 1 ▸ summarizes the final refinement statistics and geometry defined by *MolProbity* (Chen *et al.*, 2010 ▸). We prepared the structural figures using *PyMOL* (http://www.pymol.org).

### 2.4. BC hydrolysis assay

To perform the BC hydrolysis assay, we cultured *K. xylinus* to obtain BC based on the method reported previously (Czaja *et al.*, 2004 ▸). BC is porous and incorporates water molecules, forming a gelatinous structure. To reduce the variation in the produced BC, we collected BC from a cultured medium as follows. We placed *K. xylinus* cells into 5 ml Schramm–Hestrin (HS) medium (Schramm & Hestrin, 1954 ▸) and initially grew them at 30°C with stirring at 170 rev min^−1^ for four days. We then inoculated 0.1 ml cultured medium into 3 ml fresh HS medium and grew the cells at 30°C with stirring at 120 rev min^−1^ for three days. The dry mass of BC was referenced each time to control the amount of produced BC. We collected the gelatinous BC membrane and gently washed it with 3 ml buffer *D* (50 m*M* sodium citrate buffer pH 5.0 containing 200 m*M* sodium chloride). To measure BC hydrolysis, the solution containing the purified *Eb*BcsZ and *Eb*BcsZ(D242A) was changed to buffer *D*. We performed repetitive concentration and dilution of purified *Eb*BcsZ and *Eb*BcsZ(D242A) by ultrafiltration using Vivaspin 6 concentators with a 10 kDa cutoff membrane, and finally adjusted the concentration of the samples to 10 mg ml^−1^. We dissolved bovine serum albumin (BSA; Sigma–Aldrich) and cellulase ONOZUKA RS (Yakult Pharmaceutical Industry) in buffer *D* to a concentration of 10 mg ml^−1^ as negative and positive controls, respectively. We transferred the prepared gelatinous membrane of BC into 3 ml of each enzyme solution and incubated the mixtures at 30°C with stirring at 170 rev min^−1^ for 20 h. Since the turbidity of the BC solution increases as BC is hydrolyzed, we observed the turbidity of each enzyme solution to estimate the BC hydrolysis. Finally, we measured the absorbance of each sample at 600 nm. We performed four technical replicates for each condition.

## 3. Results

### 3.1. The hydrolytic activity of *Eb*BcsZ towards BC

**Figure 2:**
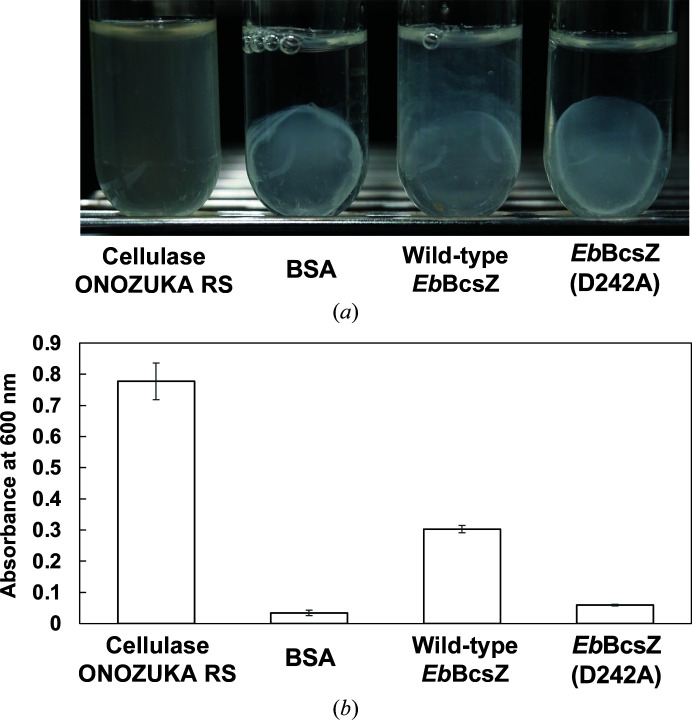
BC hydrolysis assay for *Eb*BcsZ. (*a*) The state of gelatinous BC after 20 h of incubation with cellulase ONOZUKA RS, bovine serum albumin (BSA), *Eb*BcsZ and *Eb*BcsZ(D242A). (*b*) Turbidity of the assay solution measured after 20 h of incubation. Average and standard deviation values of the absorbance at 600 nm were calculated from four replicates (*N* = 4).

Like CMCax, *Eb*BcsZ can hydrolyze cellooligosaccharides, including cellopentaose, cellohexaose and CMC, an aqueous cellulose derivative (Nakai *et al.*, 2013 ▸; Mazur & Zimmer, 2011 ▸). To investigate whether *Eb*BcsZ acts on gelatinous BC, we performed a hydrolysis assay on BC produced by *K. xylinus*. To estimate the degree of BC hydrolysis, we measured the absorbance at 600 nm representing the turbidity of BC-containing solutions in the presence or absence of *Eb*BcsZ. In the positive control, BC hydrolysis allowed the cells embedded in gelatinous BC to disperse into the solution, increasing the turbidity (Fig. 2 ▸, left). Incubating the BC-containing solution with wild-type *Eb*BcsZ partly degraded gelatinous BC (Fig. 2 ▸ *a*). The turbidity of this solution was 40% of that of the positive control (Fig. 2 ▸ *b*), indicating that *Eb*BcsZ has a moderate hydrolytic activity on BC. In contrast, the base catalyst mutant *Eb*BcsZ(D242A) barely changed the shape of gelatinous BC (Fig. 2 ▸ *a*, right), and the turbidity of the BC solution incubated with *Eb*BcsZ(D242A) was comparable to that of the negative control (Fig. 2 ▸ *b*, right).

### 3.2. The structure of *Eb*BcsZ in complex with cellooligosaccharide

**Figure 3:**
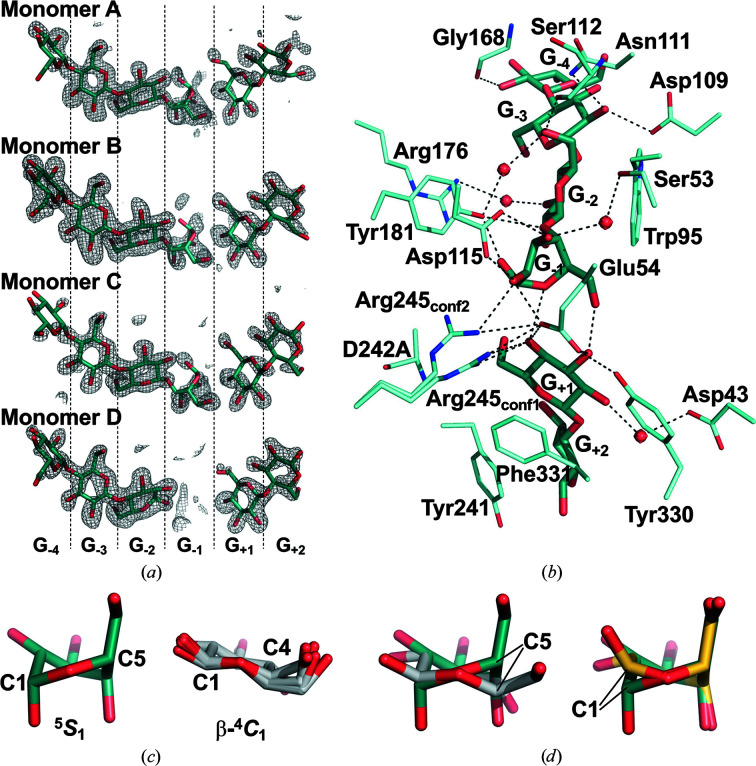
The structure of the active site in *Eb*BcsZ(D242A)_CPT_. (*a*) Electron densities of the β-1,4-glucan chains bound to the four monomers of the asymmetric unit. The electron densities are the omit map contoured at 3.0σ. (*b*) Interactions between the β-1,4-glucan chains and the residues in the active site. We selected monomer *A* as the representative active site. The water molecules and the residues interacting with the β-1,4-glucan chains are represented as red spheres and light blue lines, respectively. The hydrogen-bond network is represented as dotted lines. In (*a*) and (*b*), the β-1,4-glucan chains are shown as sticks and O, N and C atoms are shown in red, blue and dark cyan, respectively. (*c*) The conformation of G_−1_ (dark cyan) and five other glucosyl units in subsites G_−2_, G_−3_, G_−4_, G_+1_ and G_+2_ (light gray) of *Eb*BcsZ(D242A)_CPT_. (*d*) Superposed views of glucosyl units. Left: superposition of G_−1_ (dark cyan) and G_−2_ (light gray) in *Eb*BcsZ(D242A)_CPT_. Right: superposition of G_−1_ in *Eb*BcsZ(D242A)_CPT_ (dark cyan) and G_−1_ in *Ct*CelA(E95Q)_CPT_ (yellow).

Similar to the homologous enzyme *Ec*BcsZ (Mazur & Zimmer, 2011 ▸), we prepared *Eb*BcsZ without the N-terminal signal peptide (Met1–Ala20) and with a mutation of the base catalyst (D242A) to elucidate the reaction mechanism of *Eb*BcsZ. We determined the structure of *Eb*BcsZ(D242A)_CPT_ at 1.30 Å resolution by soaking *Eb*BcsZ(D242A) crystals in a solution containing CPT. In this *Eb*BcsZ(D242A)_CPT_ structure, the residues Cys22–Trp359 were visible, while the N-terminal Ala21 and the eight C-terminal residues (Gly360–Gln367) could not be built due to poor electron density. The overall structure of *Eb*BcsZ(D242A)_CPT_ formed a classical (α/α)_6_ barrel composed of six inner and lateral antiparallel helices. Although *Eb*BcsZ is a monomeric enzyme, the asymmetric unit contained four *Eb*BcsZ(D242A)_CPT_ monomers. These four monomers were essentially identical, with a root-mean-square deviation (r.m.s.d.) value of less than 0.41 Å for 335 C^α^ atoms. We selected monomer *A* for structural comparison and description of the active site. Structural comparison using *PDBeFold* (Krissinel & Henrick, 2004 ▸) showed that *Eb*BcsZ(D242A)_CPT_ was similar to the other GH8 endo-β-1,4-glucanases *Ec*BcsZ(E55Q)_CPT_ (PDB entry 3qxq), *Ct*CelA(E95Q)_CPT_ (PDB entry 1kwf) and CMCax (PDB entry 1wzz), with r.m.s.d.s of 0.65 Å for 338 C^α^ atoms, 2.09 Å for 270 C^α^ atoms and 2.02 Å for 278 C^α^ atoms, respectively. Although we soaked the crystals in a CPT-containing solution, we did not obtain full-length CPT in the *Eb*BcsZ(D242A)_CPT_ structure, unlike in the *Ec*BcsZ(E55Q)_CPT_ and *Ct*CelA(E95Q)_CPT_ structures. In each monomer of the asymmetric unit, two cellooligosaccharides occupied the plus and minus subsites (both sides of the cleavage position) of the active site (Fig. 3 ▸ *a*, Supplementary Fig. S3), indicating an intermediate state that differs from those of reported GH8 endo-β-1,4-glucanase structures. In all four monomers, cellobiose binds to subsites +1 to +2 similarly, whereas the cellooligosaccharide binds to the minus subsites differently. Cellotetraose bound to subsites −1 to −3 and extended to an additional subsite −4 in three monomers (monomers *A*–*C*), while cellotriose bound to subsites −2 to −4 in the remaining monomer (monomer *D*) (Fig. 3 ▸ *a*).

We next compared the *Eb*BcsZ(D242A)_CPT_ structure with those of other GH8 endo-β-1,4-glucanases. The cellobiose orientation in subsites +1 to +2 of *Eb*BcsZ(D242A)_CPT_ was similar to that of *Ct*CelA(E95Q)_CPT_ (Guérin *et al.*, 2002 ▸). G_+1_ and G_+2_ formed stacking interactions with the aromatic residues in the same manner. However, there were differences in the interaction between the hydroxy group of G_+1_ and polar residues in the structures of both *Eb*BcsZ(D242A)_CPT_ and *Ct*CelA(E55Q)_CPT_ (Fig. 3 ▸ *b*). Note that in the *Eb*BcsZ(D242A)_CPT_ structure the Arg245 side chain presented two different conformations (Arg245_conf1_ and Arg245_conf2_). The Arg245_conf1_ side chain interacts with the 4-OH of G_+1_. Additionally, the side chain of the general acid catalyst Glu54, corresponding to E95Q in *Ct*CelA and E55Q in *Ec*BcsZ, interacted with the 3-OH and 4-OH of G_+1_. Our structure also displayed an interaction network associated with Glu54. Glu54 interacts with Arg245 and Tyr330 through a salt bridge and a hydrogen bond, respectively. Except for G_−1_, four glucosyl units interacted with the minus subsites in the same manner as those in *Ec*BcsZ(E55Q)_CPT_ and *Ct*CelA(E95Q)_CPT_ (Mazur & Zimmer, 2011 ▸; Guérin *et al.*, 2002 ▸). In *Eb*BcsZ(D242A)_CPT_, the Asp115 side chains formed a bidentate interaction with G_−1_, and the Glu54 side chain interacted with the 2-OH of G_−1_. Furthermore, we observed an interaction between G_−1_ and G_+1_ in the *Eb*BcsZ(D242A)_CPT_ structure. The 6-OH and sugar-ring O (O5) atoms of G_−1_ interacted with the 3-OH and 4-OH of G_+1_, respectively, through hydrogen bonds.

### 3.3. The glucosyl unit conformation in subsite −1

In the *Eb*BcsZ(D242A)_CPT_ structure, G_−1_ displayed a distorted ^5^ *S*_1_ conformation, whereas the glucosyl units in the other subsites (G_−2_, G_−3_, G_−4_, G_+1_ and G_+2_) presented a stable β-^4^ *C*_1_ chair conformation (Fig. 3 ▸ *c*, Supplementary Table S1). Moreover, considering the position of the 1-OH of G_−1_, G_−1_ has the α-configuration at the C1 atom. Comparing the C5 position of G_−1_ with those of the other glucosyl units clearly shows this difference (Fig. 3 ▸ *d*, left). G_−1_ displayed a ^2,5^ *B* conformation in the *Ct*CelA(E95Q)_CPT_ structure, and comparing the C1 positions of G_−1_ in *Eb*BcsZ(D242A)_CPT_ and *Ct*CelA(E95Q)_CPT_ also shows the difference in the conformation of G_−1_ (Fig. 3 ▸ *d*, right). To confirm whether the alanine mutation of the general base catalyst Asp242 causes structural changes, we determined the structure of *Eb*BcsZ in complex with glycerol (*Eb*BcsZ_GOL_). We obtained *Eb*BcsZ crystals in the same crystal form as those of *Eb*BcsZ(D242A)_CPT_. Residues Cys22–Trp359 of each *Eb*BcsZ_GOL_ monomer were built in the asymmetric unit. *Eb*BcsZ_GOL_ and *Eb*BcsZ(D242A)_CPT_ had very similar structures, with an r.m.s.d. of 0.17 Å for 335 C^α^ atoms, demonstrating that the D242A mutation scarcely changed the overall *Eb*BcsZ structure. In the *Eb*BcsZ_GOL_ structure, subsites −1, −2 and +1 were occupied by glycerol molecules used as a cryoprotectant for the diffraction experiment (Supplementary Fig. S4*a* ). These glycerol molecules can be considered to be a mimic of the bound glucosyl unit of β-1,4-glucan. In each *Eb*BcsZ monomer the glycerol molecule in subsite −1 adopted a remarkably similar orientation. Superposing *Eb*BcsZ_GOL_ on *Eb*BcsZ(D242A)_CPT_ showed that three hydroxy groups of the glycerol and two water molecules were close to the O5 atom and 3-OH, 6-OH, 1-OH and 2-OH of G_−1_, respectively (Supplementary Fig. S4*b* , Supplementary Table S2), supporting the plausibility of the formation of a distorted ^5^ *S*_1_ conformation of G_−1_ during the hydrolytic reaction process. Besides, the existence of the ^5^ *S*_1_ conformation is consistent with the predicted result of a quantum mechanics/molecular mechanics (QM/MM) dynamic simulation on the reaction process of GH8 enzymes (Ardèvol & Rovira, 2015 ▸; Petersen *et al.*, 2009 ▸).

### 3.4. The secondary binding site of the β-1,4-glucan chain

**Figure 4:**
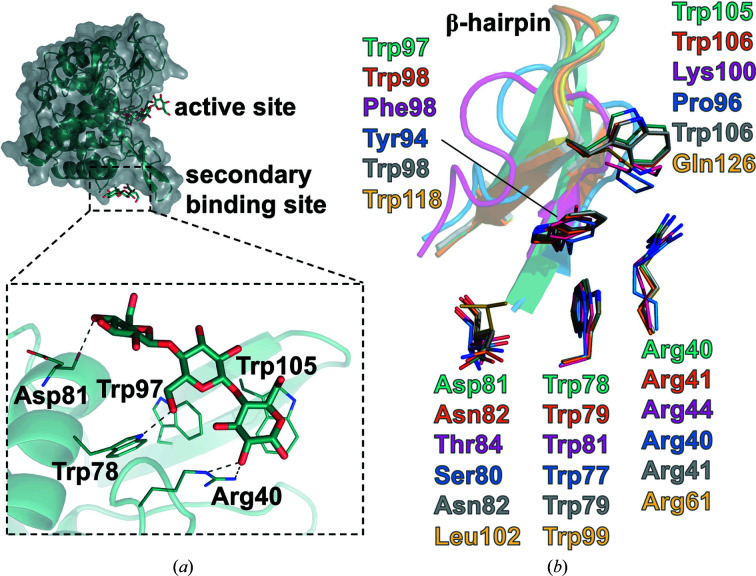
The structure of the secondary binding site in *Eb*BcsZ(D242A)_CPT_. (*a*) Close-up view of the secondary binding site. The cellotriose and the residues interacting with it are represented as sticks and lines, respectively. The hydrogen bonds between cellotriose and the residues of *Eb*BcsZ(D242A)_CPT_ are represented as dotted lines. (*b*) Superposition of the residues interacting with cellotriose in *Eb*BcsZ and the other endo-β-1,4-glucanases. The residues of *Eb*BcsZ(D242A)_CPT_ (dark cyan), *Ec*BcsZ(E55Q)_CPT_ (PDB entry 3qxq, orange), CMCax (PDB entry 1wzz, pink), Cel10 (PDB entry 5gy3, blue), BcsZ (PDB entry 4q2b, gray) and hydrolase from *Vibrio fischeri* (PDB entry 5cd2, yellow) are labeled and represented as lines. The β-hairpin region of *Eb*BcsZ(D242A)_CPT_ and the corresponding regions in the other endo-β-1,4-glucanases are represented as ribbons.

Among the GH8 endo-β-1,4-glucanases, the structure of *Eb*BcsZ(D242A)_CPT_ is the first to show that β-1,4-glucan binds to the surface away from the active site (Fig. 4 ▸ *a*), which is called the secondary binding site (Cuyvers *et al.*, 2012 ▸). Two of the four *Eb*BcsZ(D242A)_CPT_ monomers (monomers *A* and *C*) bind to cellotriose through a tryptophan-rich cleft (Fig. 4 ▸ *a*). However, electron density for the cellooligosaccharide did not appear in the corresponding region in monomers *B* and *D*, which can be considered to be an effect of crystal packing (Supplementary Fig. S5). This marked difference implies that the physical and chemical environment easily affects the binding of β-1,4-glucan at the secondary binding site. Cellotriose bound to monomers *A* and *C* in the same manner. The glucosyl units at the central part and the reducing end formed a T-shaped stacking with Trp97 and π–π stacking with Trp105, respectively. Stacking interactions with aromatic residues are common in secondary binding sites and seem to be essential for oligosaccharide binding (Cockburn *et al.*, 2016 ▸; Cockburn & Svensson, 2013 ▸; Cuyvers *et al.*, 2011 ▸). The 2-OH of the reducing-end sugar and the 6-OH of the central sugar interacted with the side chains of Arg40 and Trp78, respectively. The 4-OH of the non-reducing-end sugar interacted with the main-chain carbonyl of Asp81 through a hydrogen bond. These contacts are probably conserved among the other GH8 endo-β-1,4-glucanases, except for the stacking with Trp105 in a β-hairpin motif (Fig. 4 ▸ *b*).

## 4. Discussion

BC, which is generated by cellulose-producing bacteria, is a bio­material with remarkable characteristics that are superior to those of the cellulose fiber extracted from plants. It has potential applications in various fields. In BC-producing bacteria, the endo-β-1,4-glucanase serves as a cleaner protein that degrades disordered cellulose fibers, efficiently producing high-quality BC. Our work is focused on investigating the structural and functional properties of the endo-β-1,4-glucanase BcsZ in order to help to improve BC production.

A BC degradation assay showed that *Eb*BcsZ has hydrolytic activity, although it is weaker than that of cellulase ONOZUKA RS, a multi-component cellulase. This weak activity may arise from limited degradation of the disordered region in gelatinous BC, which is in agreement with previous studies showing that *Eb*BcsZ can degrade cellooligosaccharides and CMC to noncrystalline cellulose (Koo *et al.*, 1998 ▸). Considering that the endo-β-1,4-glucanase *Eb*BcsZ needs to access the β-1,4-glucan chain to cleave internal bonds, secondary binding sites probably assist in the binding of the target substrate β-1,4-glucan chain. Moreover, immunostaining of CMCax (Standal *et al.*, 1994 ▸) and digestion of BcsZ from *Salmonella typhimurium* by a protease (Ahmad *et al.*, 2016 ▸) demonstrated that these endo-β-1,4-glucanases are located close to the outer membrane or periplasmic space, allowing them to degrade β-1,4-glucan chains before crystallization (Ahmad *et al.*, 2016 ▸; Hu *et al.*, 2010 ▸; Standal *et al.*, 1994 ▸). A phylogenetic relationship shows that the gene encoding endo-β-1,4-glucanase appears with the *bcsC* gene in the *bcs* gene cluster, with some exceptions (Römling & Galperin, 2015 ▸). This little-studied BcsC subunit of TC localizes at the outer membrane and periplasmic space, and contributes to the export of BC to the extracellular matrix. Thus, endo-β-1,4-glucanase may be associated with BcsC to access noncrystalline β-1,4-glucan chains efficiently.

**Figure 5:**
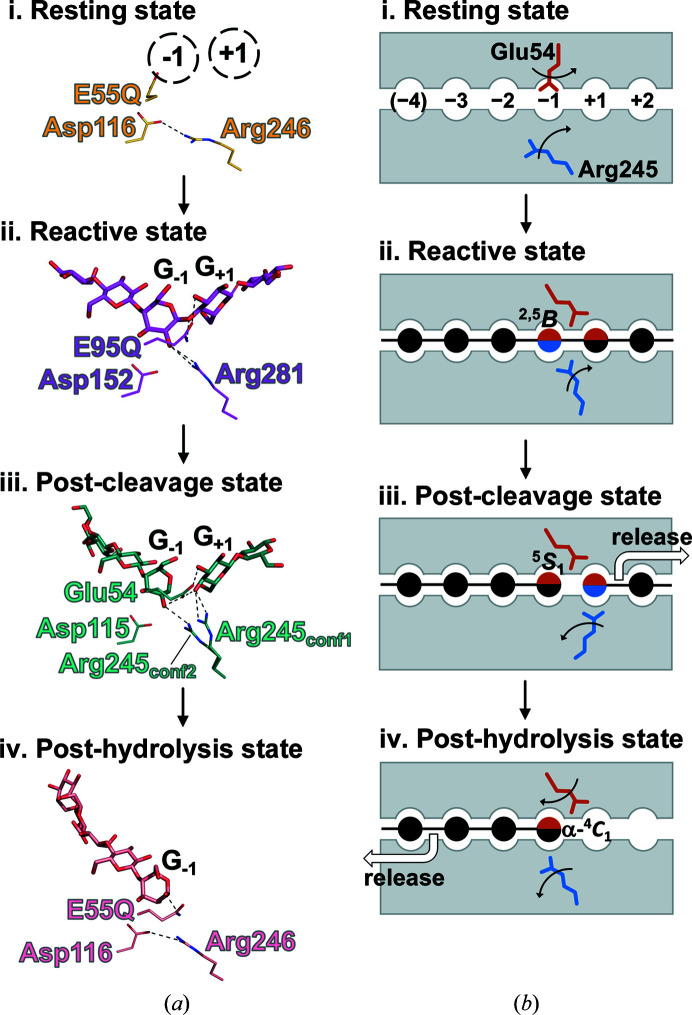
Interactions between the bound β-1,4-glucan chains and the polar residues of GH8 endo-β-1,4-glucanases in each reaction state. (*a*) The structures of (i) the resting state *Ec*BcsZ_apo_, (ii) the reactive state *Ct*CelA(E95Q)_CPT_, (iii) the post-cleavage state *Eb*BcsZ(D242A)_CPT_ and (iv) the post-hydrolysis state *Ec*BcsZ(E55Q)_CPT_. The cellooligosaccharides and the residues are represented as sticks and lines, respectively. (*b*) Schematic diagram of the motion of the GH8 endo-β-1,4-glucanase residues during the reaction process. The glutamate and arginine of interest are represented as orange and blue sticks, respectively. When the sugar units interact with the glutamate and/or arginine, the solid circles are in the same color as the glutamate and/or arginine.

Several studies have reported structures of wild-type GH8 endo-β-1,4-glucanases and general acid catalyst mutants. These studies considered that the apo-form structures of these GH8 endo-β-1,4-glucanases represented the ‘resting state’. Here, we select *Ec*BcsZ_apo_ as representing the resting state (Fig. 5 ▸ *a*, i) since it is most similar to *Eb*BcsZ in both sequence and structure. In the structures of *Ct*CelA(E95Q)_CPT_ (Guérin *et al.*, 2002 ▸) and *Tt*GH8_XTO_ (Fowler *et al.*, 2018 ▸), a single oligosaccharide was incorporated into the subsites, and G_−1_ and X_−1_ showed a distorted ^2,5^ *B* conformation. These complex structures represent the ‘reactive state’ that readily forms the oxocarbenium intermediate (Fig. 5 ▸ *a*, ii). In the *Ec*BcsZ(E55Q)_CPT_ structure cellopentaose was only located in the minus subsites, meaning that *Ec*BcsZ(E55Q)_CPT_ represents the ‘post-hydrolysis state’ that occurs after release of the leaving group (Fig. 5 ▸ *a*, iv). We employed a base-catalyst mutant *Eb*BcsZ(D242A) and determined its cellooligosaccharide-bound structure, unlike previous studies. The *Eb*BcsZ(D242A)_CPT_ structure provides a novel snapshot of the reaction state, in which two β-1,4-glucan chains lying in subsites −1 to −4 and +1 to +2 are separated. The distance between the C1 atom of G_−1_ and the 4-OH of G_+1_ is approximately 3 Å, which is longer than the typical length of a C—O single bond (1.43 Å). Thus, *Eb*BcsZ(D242A)_CPT_ represents the ‘post-cleavage state’, with G_−1_ in a distorted ^5^ *S*_1_ conformation, immediately after the scissile-bond cleavage (Fig. 5 ▸ *a*, iii).

The residues Glu54 and Arg245 in *Eb*BcsZ(D242A)_CPT_ differed in orientation compared with the corresponding residues in *Ec*BcsZ(E55Q)_CPT_ (E55Q and Arg246) and *Ct*CelA(E95Q)_CPT_ (E95Q and Arg281). Similar to the reactive state [*Ct*CelA(E95Q)_CPT_] and the post-hydrolysis state [*Ec*BcsZ(E55Q)_CPT_], the side chain of the acid catalyst Glu54 was located between G_+1_ and G_−1_ in the post-cleavage state [*Eb*BcsZ(D242A)_CPT_] (Fig. 5 ▸ *a*, ii–iv). However, comparison between *Eb*BcsZ(D242A)_CPT_ and *Ec*BcsZ_apo_ indicated that when the substrate binds, the Glu54 side chain would simultaneously flip and interact with Tyr330 and Arg245 to avoid a clash with the 3-OH of G_−1_ (Figs. 3 ▸ *b* and 5 ▸ *a*, i). Thus, upon substrate binding, the acid catalyst side chain flips and keeps this conformation, while the cellooligosaccharide occupies subsite −1. In the resting state (*Ec*BcsZ_apo_), the N^η1^ atom of Arg246 forms a salt bridge to the O^δ2^ atom of Asp116 and was distant from the binding subsites (Fig. 5 ▸ *a*, i). In the reactive state [*Ct*CelA(E95Q)_CPT_], the corresponding Arg281 was distant from Asp152 and shifted to interact with G_−1_ (Fig. 5 ▸ *a*, ii). In the post-cleavage state [*Eb*BcsZ(D242A)_CPT_], Arg245 presented two conformations: Arg245_conf2_ was the same as that of the corresponding Arg281 of *Ct*CelA in the reactive state, whereas Arg245_conf1_ shifted to interact with G_+1_ (Figs. 3 ▸ *b* and 5 ▸ *a*, iii). In the post-hydrolysis state [*Ec*BcsZ(E55Q)_CPT_], Arg246 returned to interact with Asp116 and was distant from subsite +1 (Fig. 5 ▸ *a*, iv). Therefore, this arginine residue has an important role in tethering the cleaved β-1,4-glucan chains. Based on these findings, we summarized a series of conformational changes in the catalytic process of GH8 endo-β-1,4-glucanase to illustrate the whole catalytic mechanism (Fig. 5 ▸ *b*). The flexibility of the arginine residue allows the post-cleavage state to be captured. The superimposed structures of *Eb*BcsZ(D242A)_CPT_ and *Eb*BcsZ_GOL_ clearly showed that the side chain of Asp242 does not sterically hinder the conformational changes of G_−1_ and Arg245 (Supplementary Fig. S6). Considering the reaction mechanism of an inverting glycoside hydrolase (Fig. 1 ▸), the acid catalyst mutation prevents the formation of the transition intermediate, and the ^5^ *S*_1_ conformation is invisible. The base catalyst activates a water molecule for a nucleophilic attack on the C1 atom of G_−1_ and does not directly attack G_−1_. Even though the base catalyst is mutated, the reaction may proceed by the very weak nucleophilicity of water. Indeed, previous studies demonstrated that mutating the base catalyst drastically reduced, but did not completely remove, the activity of GH8 enzymes (Fowler *et al.*, 2018 ▸; De Vos *et al.*, 2006 ▸; Collins *et al.*, 2005 ▸). The structural comparison of G_−1_ in the post-cleavage state ^5^ *S*_1_ [*Eb*BcsZ(D242A)_CPT_] with the substrate state β-^4^ *C*_1_ [the other glucosyl unit in *Eb*BcsZ(D242A)_CPT_] and the transition state ^2,5^ *B* [*Ct*CelA(E95Q)_CPT_] clearly showed the difference in planarity of G_−1_ (Supplementary Fig. S3*c* ). The boat ^2,5^ *B* conformer changes to a skew-boat ^5^ *S*_1_ conformer, indicating that the hydrolysis reaction of GH8 enzymes precedes via an unstable conformation to meet the requirements of an antiperiplanar lone-pair hypothesis (Nerinckx *et al.*, 2005 ▸).

**Figure 6:**
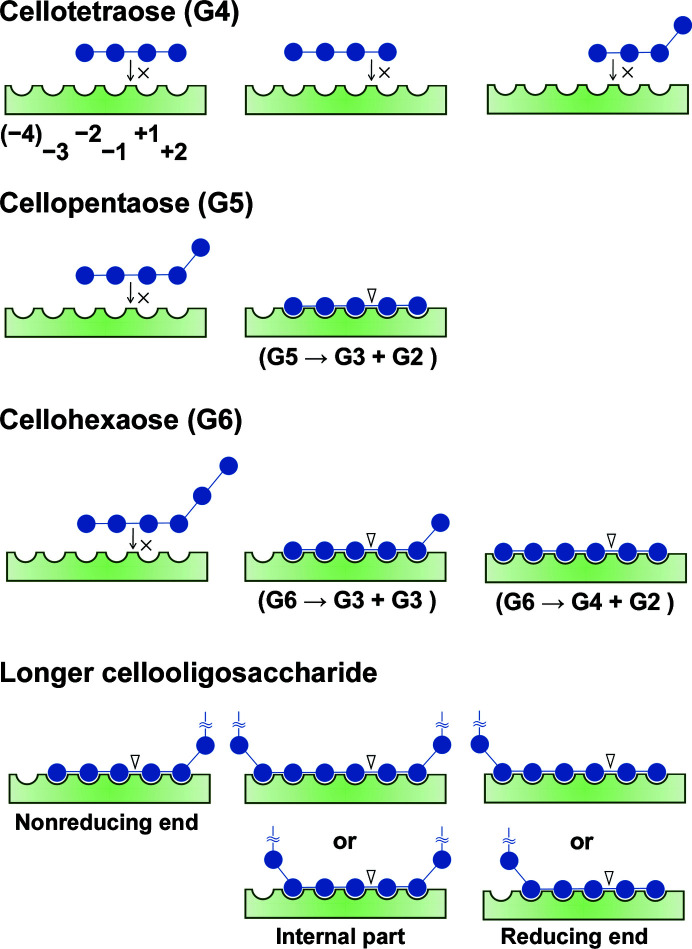
Schematic diagram of the proposed binding patterns of oligosaccharides in BcsZ. The β-1,4-glucan chains cellotetraose (G4), cellopentaose (G5), cellohexaose (G6) and longer cellooligosaccharides are represented as solid spheres connected by lines. The triangle indicates the scissile bond.

In the structure of *Eb*BcsZ(D242A)_CPT_, we obtained electron density for six glucosyl units in monomers *A*–*C* as shown in Fig. 3 ▸(*a*), although we utilized cellopentaose. Considering that no electron density appeared at subsite −1 in monomer *D* and the electron density for G_−1_ in monomers *A*–*C* clearly showed the ^5^ *S*_1_ conformation, the electron density might be derived from a mixture of the post-cleavage state of cellopentaose in subsites −3 to +2 and cleaved cellotriose in subsites −4 to −2, but not noncleaved cellopentaose in subsites −4 to +1. Combining the binding of oligosaccharides in the structure of *Eb*BcsZ(D242A)_CPT_ with a previous thin-layer chromatography (TLC) analysis (Sunagawa *et al.*, 2012 ▸), we proposed the binding patterns of oligosaccharides in the trimming mechanism of BcsZ (Fig. 6 ▸). The structure shows that *Eb*BcsZ has six subsites, −3 to +2 and additionally −4, for binding the β-1,4-glucan chain. The TLC analysis demonstrated that *Eb*BcsZ degrades cellopentaose into cellobiose and cellotriose but does not degrade cellotetraose. Thereby, five cellopentaose glucosyl units should lie in subsites −3 to +2 and not in subsites −4 to +1 or in the four subsites −2 to +2 (Fig. 6 ▸, G4 and G5). The TLC analysis also showed that *Eb*BcsZ degrades cellohexaose into two cellotrioses or into cellobiose and cellotetraose. Cellohexaose needs to have five glucosyl units in subsites −3 to +2 and a free glucosyl unit to produce two cellotrioses. The six glucosyl units of cellohexaose need to be in subsites −4 to +2 (Fig. 6 ▸, G6) to obtain cellobiose and cellotetraose. Consequently, *Eb*BcsZ hydrolyzes cellopentaose or longer cellooligosaccharides when the β-1,4-glucan chain occupies at least subsites −3 to +2 (Fig. 6 ▸, bottom). Moreover, considering that glycerol molecules preferentially bind to subsites −2 to +1 in the *Eb*BcsZ_GOL_ structure (Supplementary Fig. S3*c* ), the sugar units in subsites −3 and +2 might have a relatively lower affinity than those in subsites −2 to +1. Therefore, subsites −3 and +2 assist in binding the β-1,4-glucan chain.

## 5. Conclusion

This work demonstrated that the GH8 endo-β-1,4-glucanase *Eb*BcsZ partly degrades gelatinous BC, implying that it plays a potential role in the quality control of BCs. Unlike previous studies, we obtained the *Eb*BcsZ(D242A)_CPT_ structure using a base-catalyst mutant, revealing a ^5^ *S*_1_ intermediate as the ‘post-cleavage state’. By comparing GH8 endo-β-1,4-glucanase snapshots, we provided insights into the motion of the residues coupled to the conformational change of the glucosyl unit in subsite −1 during the reaction process. Moreover, based on structural analysis of *Eb*BcsZ(D242A)_CPT_, we proposed a β-1,4-glucan-trimming mechanism for *Eb*BcsZ to enhance the understanding of the biosynthesis of high-quality cellulose by bacteria, and our findings open up a route to a deep understanding of the enzymology of glycoside hydrolases.

## Supplementary Material

PDB reference: *Eb*BcsZ bound to glycerol, 7f81

PDB reference: *Eb*BcsZ bound to cellooligosaccharide, 7f82

Supplemental information. DOI: 10.1107/S2059798321012882/ji5024sup1.pdf

## Figures and Tables

**Table 1:** Data-collection and refinement statistics Values in parentheses are for the highest resolution shell.

	*Eb*BcsZ(D242A)_CPT_	*Eb*BcsZ_GOL_
Data collection		
Wavelength (Å)	1.0000	1.0000
Space group	*P*2_1_	*P*2_1_
*a*, *b*, *c* (Å)	89.3, 92.9, 90.5	89.3, 91.5, 89.9
α, β, γ (°)	90, 98.4, 90	90, 98.2, 90
Resolution (Å)	50.0–1.30 (1.38–1.30)	50.0–1.93 (2.05–1.93)
*R*_merge_ †	0.030 (0.567)	0.063 (0.597)
Multiplicity	4.2 (3.5)	3.8 (3.8)
Completeness (%)	99.5 (98.8)	99.7 (99.3)
No. of unique reflections	355775 (57751)	107156 (17177)
Mean *I*/σ(*I*)	21.4 (2.3)	18.3 (2.6)
CC_1/2_	1.000 (0.873)	0.999 (0.837)
Refinement		
Resolution (Å)	42.51–1.30	38.34–1.93
*R*_work_ ‡/*R*_free_ §	0.169/0.187	0.162/0.197
No. of atoms		
Protein	10990	10876
Water	1179	957
Cellooligosaccharide	329	—
Glycerol	—	126
Disodium tartrate	20	20
R.m.s.d.s		
Bond lengths (Å)	0.014	0.009
Bond angles (°)	1.573	0.952
Ramachandran statistics (%)		
Favored	98.59	98.51
Allowed	1.41	1.49
Outliers	0	0
Mean *B* factors (Å^2^)		
Protein	24.57	30.69
Water	33.01	38.22
Cellooligosaccharide	32.26	—
Glycerol	—	38.99
Disodium tartrate	22.11	26.78
PDB code	7f82	7f81

† *R*_merge_ = \textstyle \sum_{hkl}\sum_{i}|I_{i}(hkl)- \langle I(hkl)\rangle|/\textstyle \sum_{hkl}\sum_{i}I_{i}(hkl), where *i* is the number of observations of a given reflection and 〈*I*(*hkl*)〉 is the average intensity of the *i* observations.

‡ *R*_work_ = \textstyle \sum_{hkl}\big ||F_{\rm obs}|-|F_{\rm calc}|\big |/\textstyle \sum_{hkl}|F_{\rm obs}|.

§ *R*_free_ was calculated using a randomly selected 5% of reflections that were excluded from refinement.
