# Supplementary material for: Structural snapshot of a glycoside hydrolase family 8 endo-β-1,4-glucanase capturing the state after cleavage of the scissile bond
Source: Acta Crystallogr D Struct Biol. 2022 Jan 24;78(Pt 2):228–37. doi: 10.1107/S2059798321012882 (PMC8805304; doi:10.1107/S2059798321012882)
Supplement: Supplementary file 1 [file d-78-00228-sup1.pdf]

STRUCTURAL
BIOLOGY

Volume 78 (2022)

Supporting information for article:

Structural snapshot of a glycoside hydrolase family 8 endo-β-1,4-glucanase capturing the state after cleavage of the scissile bond

Takaaki Fujiwara, Ayumi Fujishima, Yui Nakamura, Kenji Tajima and Min Yao

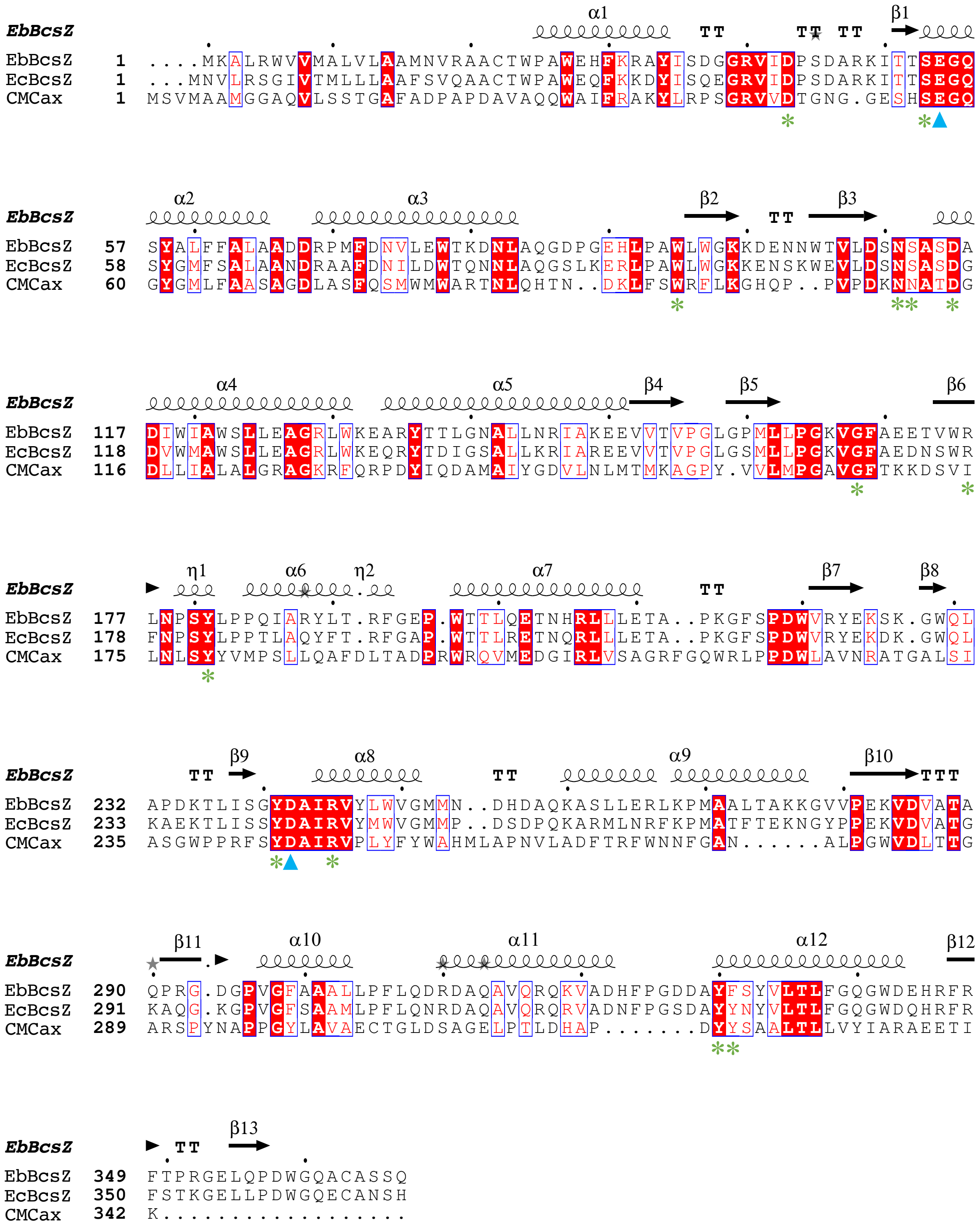

Figure S1. Multiple sequence alignment of *Eb*BcsZ (GenBank: BAM44856.1), *Ec*BcsZ (GenBank: AAB76556.1), and CMCax (GenBank: AAA16969.1). The asterisks (green) and triangles (blue) represent the residues located around the catalytic site of *Eb*BcsZ and catalytic residues, respectively.

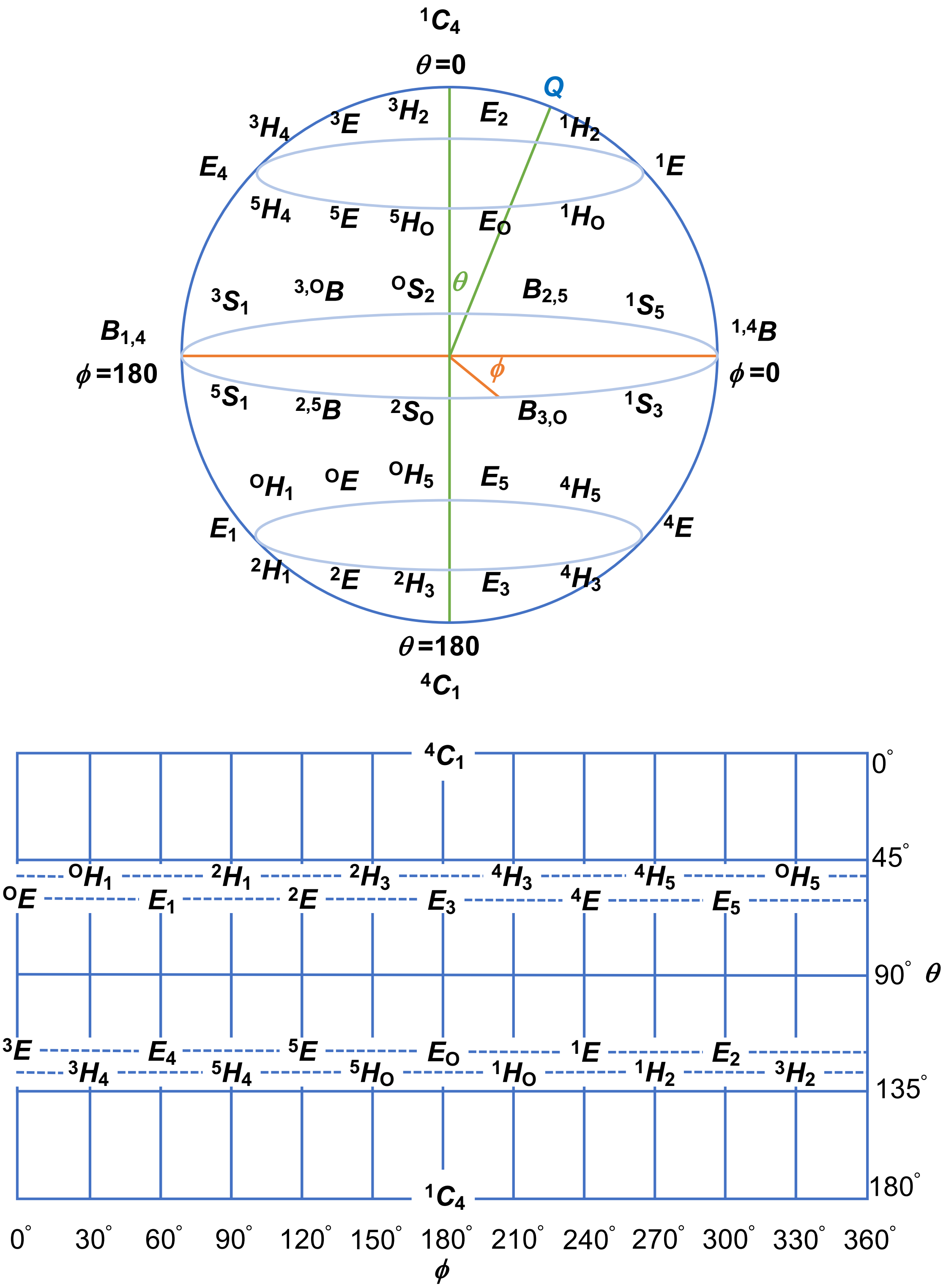

Figure S2. Conformation of pyranose represented by Cremer-Pople sphere (upper) and Mercator representation (lower). Cremer-Pople parameters ϕ, θ, and Q are also displayed (Cremer and Pople, 1975). The conformation of pyranose is represented by the puckering parameters, ϕ, θ, and Q, which shows 38 different conformers.

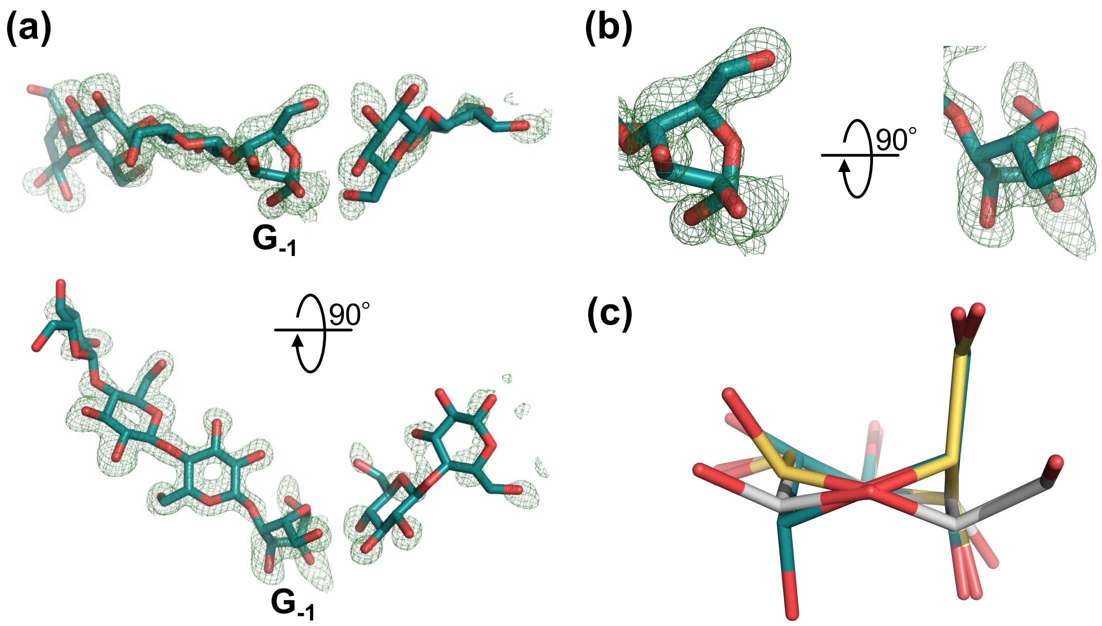

Figure S3. The structures of cellooligosaccharides bound to monomer A in *Eb*BcsZ(D242A)$_{CPT}$. (a) The omit map of cellooligosaccharides contoured at 3.0 σ. (b) Close-up view of G_{-1} in (a). (c) Superposed view of glucosyl units. Superposition of G_{-1} (green) and G_{-2} (light gray) in *Eb*CeSZ(D242A)$_{CPT}$ and G_{-1} in *Ct*CelA(E95Q)$_{CPT}$ (yellow).

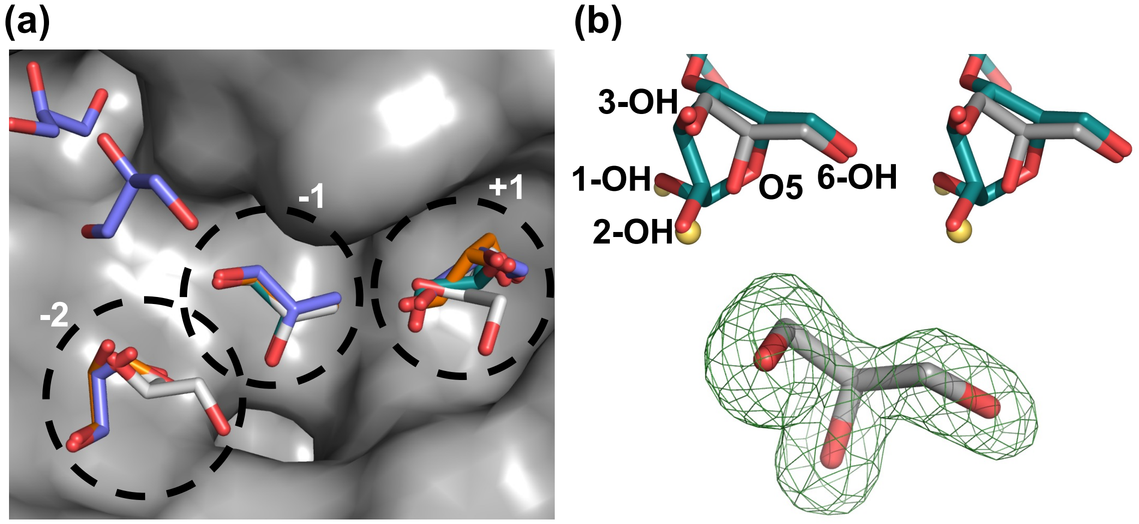

Figure S4. Glycerols bound to the active site in *Eb*BcsZ. (a) The conformation of bound glycerols around the subsites −2, −1, and +1. The glycerols bound to monomers A, B, C, and D of the asymmetric unit were superposed and shown in green, gray, purple, and orange, respectively. Oxygen atoms are red. (b) Stereo view of the superposition of G_{-1} (dark green) and glycerol (gray) in the subsite −1 (upper). Water molecules overlapped on the 1-OH and 2-OH of G_{-1} appear as yellow spheres. Oxygen atoms are red. The close up view of glycerol located at the subsite −1 of monomer A with the omit map contoured at 4.0 σ (lower).

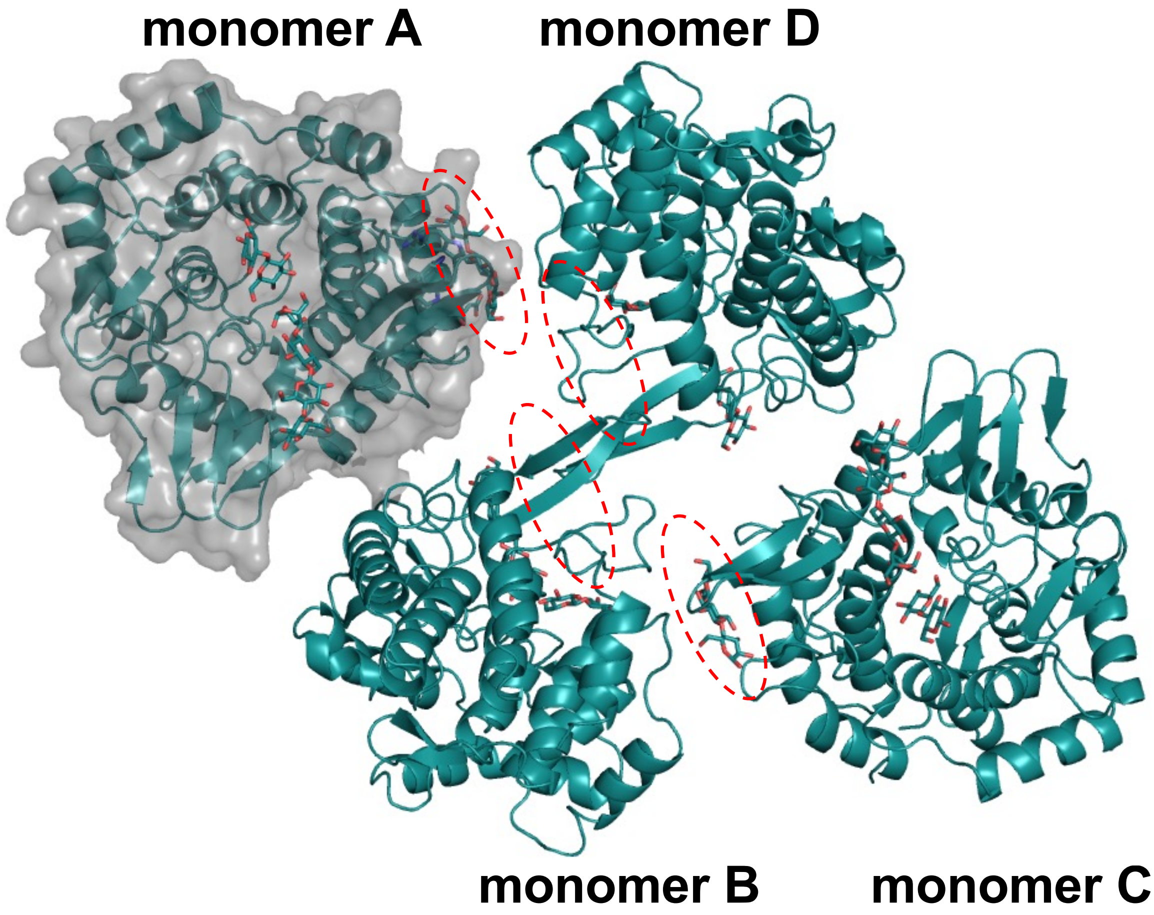

Figure S5. The molecules in the asymmetric unit of *Eb*BcsZ(D242A)$_{CPT}$. Dotted lines (red) represent the secondary binding site of each monomer in the asymmetric unit. The cellooligosaccharides appear as sticks.

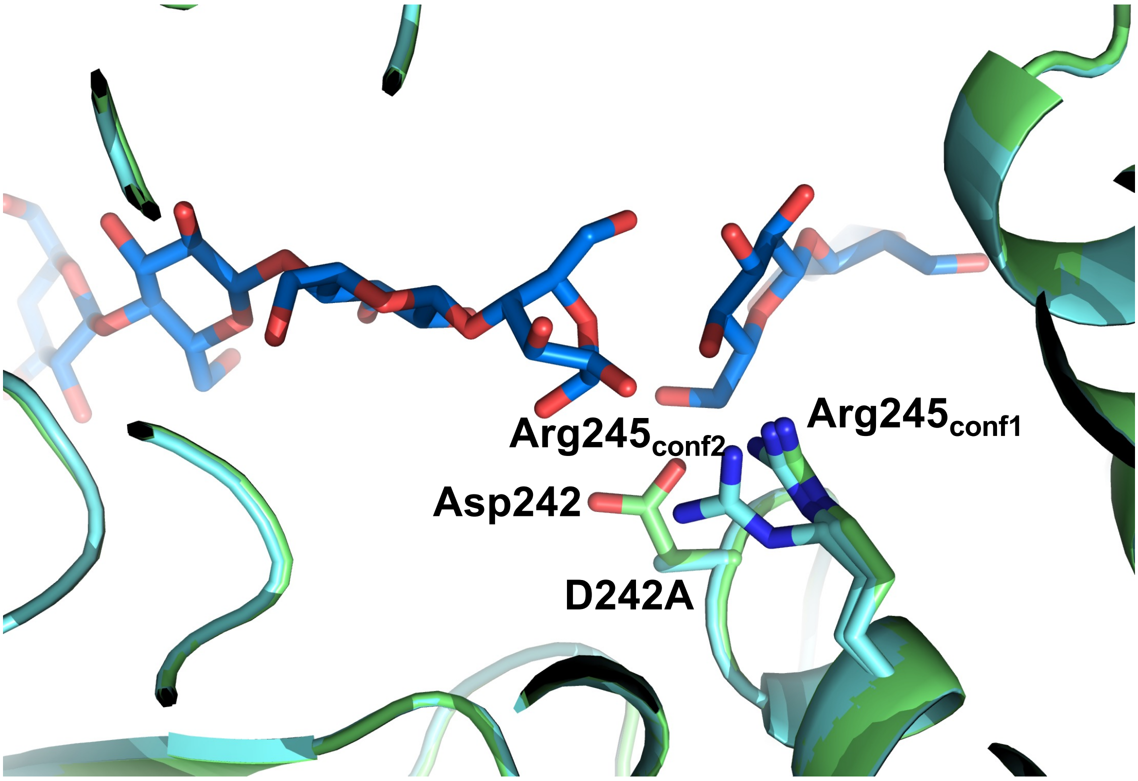

Figure S6. Superposed structures of *Eb*BcsZ(D242A)$_{CPT}$ and *Eb*BcsZ$_{GOL}$. *Eb*BcsZ(D242A)$_{CPT}$ (light blue) and *Eb*BcsZ$_{GOL}$ (light green) represented as ribbon. The cellooligosaccharides (blue) and the residues (Asp242, D242A, and Arg245) are represented as sticks.

Table S1. Cremer-Pople parameters

monomer	subsite	Cremer-Pople parameters			conformation
		ϕ (°)	θ (°)	Q (Å)	
A	−4	30.6	7.8	0.54	$^{4}C_{1}$
	−3	306.6	7.5	0.57	$^{4}C_{1}$
	−2	38.0	4.1	0.61	$^{4}C_{1}$
	−1	97.6	78.3	0.81	$^{5}S_{1}$
	+1	355.2	1.9	0.60	$^{4}C_{1}$
	+2	82.9	27.0	0.51	$^{4}C_{1}$
B	−4	307.9	8.1	0.58	$^{4}C_{1}$
	−3	315.3	8.1	0.57	$^{4}C_{1}$
	−2	355.2	7.0	0.64	$^{4}C_{1}$
	−1	95.8	85.0	0.74	$^{5}S_{1}$
	+1	278.0	7.1	0.62	$^{4}C_{1}$
	+2	79.1	2.8	0.57	$^{4}C_{1}$
C	−4	10.5	8.1	0.51	$^{4}C_{1}$
	−3	303.1	7.2	0.62	$^{4}C_{1}$
	−2	54.2	6.2	0.58	$^{4}C_{1}$
	−1	99.1	81.9	0.82	$^{5}S_{1}$
	+1	234.6	12.6	0.57	$^{4}C_{1}$
	+2	23.4	4.4	0.55	$^{4}C_{1}$
D	−4	73.8	3.5	0.52	$^{4}C_{1}$
	−3	319.4	5.7	0.61	$^{4}C_{1}$
	−2	357.7	4.2	0.59	$^{4}C_{1}$
	+1	154.6	2.0	0.57	$^{4}C_{1}$
	+2	106.7	4.0	0.55	$^{4}C_{1}$

Table S2. Distances between oxygen atoms of glycerols and waters in *Eb*BcsZ$_{GOL}$ and five oxygen atoms of G$_{-1}$ in *Eb*BcsZ(D242A)$_{CPT}$

O1 (1-OH)			O2 (2-OH)		
*Eb*BcsZ$_{GOL}$	*Eb*BcsZ(D242A)$_{CPT}$	distance (Å)	*Eb*BcsZ$_{GOL}$	*Eb*BcsZ(D242A)$_{CPT}$	distance (Å)
monomer A	monomer A	0.96	monomerA	monomer A	0.22
	monomer B	0.68		monomer B	0.84
	monomer C	0.84		monomer C	0.20
monomer B	monomer A	1.05	monomer B	monomer A	0.20
	monomer B	0.75		monomer B	0.94
	monomer C	0.93		monomer C	0.20
monomer C	monomer A	0.97	monomer C	monomer A	0.23
	monomer B	0.69		monomer B	0.81
	monomer C	0.84		monomer C	0.15
monomer D	monomer A	0.98	monomer D	monomer A	0.08
	monomer B	0.67		monomer B	0.84
	monomer C	0.86		monomer C	0.06
		0.85 ± 0.13			0.40 ± 0.34

O3 (3-OH)			O5		
*Eb*BcsZ$_{GOL}$	*Eb*BcsZ(D242A)$_{CPT}$	distance (Å)	*Eb*BcsZ$_{GOL}$	*Eb*BcsZ(D242A)$_{CPT}$	distance (Å)
monomer A	monomer A	0.39	monomer A	monomer A	1.04
	monomer B	0.42		monomer B	1.41
	monomer C	0.37		monomer C	1.17
monomer B	monomer A	0.34	monomer B	monomer A	0.92
	monomer B	0.33		monomer B	1.29
	monomer C	0.31		monomer C	1.04
monomer C	monomer A	0.36	monomer C	monomer A	0.87
	monomer B	0.35		monomer B	1.23
	monomer C	0.32		monomer C	0.99
monomer D	monomer A	0.33	monomer D	monomer A	0.92
	monomer B	0.35		monomer B	1.29
	monomer C	0.29		monomer C	1.05
		0.35 ± 0.04			1.10 ± 0.17

O6 (6-OH)		
*Eb*BcsZ$_{GOL}$	*Eb*BcsZ(D242A)$_{CPT}$	distance (Å)
monomer A	monomer A	0.24
	monomer B	0.16
	monomer C	0.23
monomer B	monomer A	0.26
	monomer B	0.17
	monomer C	0.31
monomer C	monomer A	0.22
	monomer B	0.14
	monomer C	0.26
monomer D	monomer A	0.27
	monomer B	0.17
	monomer C	0.33
		0.23 ± 0.06
